# Bioactive Phenolic Compounds in Extra Virgin Olive Oil: Implications for Cardiovascular Health

**DOI:** 10.1002/fsn3.71441

**Published:** 2026-02-13

**Authors:** Cristina Vázquez‐Jiménez, Mª. Dolores Rodríguez‐Pérez, Laura Ortega‐Hombrados, Ana María Sánchez‐Tévar, José Pedro de la Cruz‐Cortés, José A. Gonzalez‐Correa

**Affiliations:** ^1^ Departamento de Farmacología, Instituto de Investigación Biomédica de Málaga y Plataforma en Nanomedicina‐IBIMA Plataforma BIONAND, Facultad de Medicina Universidad de Málaga Málaga Spain

## Abstract

Polyphenols, bioactive compounds abundant in plant‐based foods, have attracted significant interest for their potential cardiovascular benefits. This narrative review summarizes the current evidence on how polyphenols contained in extra virgin olive oil impact cardiovascular health, including their molecular mechanisms of action and clinical effects. Polyphenols exert antioxidant and anti‐inflammatory effects in vascular cells by modulating key signaling pathways (e.g., NF‐κB, Nrf2, and PI3K/Akt) and activating endothelial nitric oxide production, which collectively may improve endothelial function and reduce atherosclerotic burden. We review human trials of polyphenol‐rich foods (such as berries, cocoa, tea, and wine) and isolated polyphenol supplements, which generally report improvements in blood pressure, vascular function, lipid profiles, and inflammatory markers—though results are not uniform. Limitations of these trials (small sample sizes, short durations) and variability in individual responses are discussed. We also consider the role of polyphenol metabolism and bioavailability, noting that gut microbiota–derived metabolites (e.g., urolithins, equol) likely contribute to the cardioprotective effects. Overall, a diet rich in diverse polyphenols appears to confer cardiovascular benefits, but more personalized research is needed to define optimal types and doses for specific patient profiles. Practical recommendations for incorporating polyphenol‐rich foods into cardiovascular prevention strategies are provided.

## Graphical abstract

Extra virgin olive oil (EVOO) is rich in bioactive polyphenols that exert antioxidant, anti‐inflammatory, and anti‐atherogenic effects. These compounds improve endothelial function, modulate lipid metabolism, and reduce platelet aggregation, contributing to cardiovascular protection. Regular consumption of phenolic‐rich EVOO, as part of a Mediterranean diet, supports vascular health and may lower the risk of cardiovascular disease through multiple molecular pathways involving NF‐κB, Nrf2, and nitric oxide signaling.

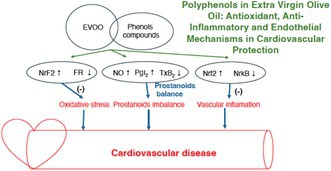

## 1 Justification and Objectives

Interest in polyphenols and their impact on cardiovascular health has grown exponentially, as reflected by an increasing number of clinical and epidemiological studies exploring their benefits. Given the vast amount of available information, there is a pressing need to complement observational findings with well‐controlled clinical studies that validate their effects in real‐world settings. While large trials such as the PREDIMED study have demonstrated the relevance of these compounds in cardiovascular disease prevention, further rigorous clinical research is required to consolidate the evidence.

This article critically reviews the most recent studies, focusing on the mechanisms of action of polyphenols, particularly those found in extra virgin olive oil (EVOO), and their impact on key cardiovascular biomarkers. Despite existing limitations regarding the availability of large‐scale and well‐controlled clinical trials, this review highlights the potential of polyphenols as a therapeutic dietary strategy and underscores the need for continued research into their role in cardiovascular health.

The objective of this narrative review is to analyze the benefits of bioactive compounds in olive oil, with a particular emphasis on polyphenols and their mechanisms of action in cardiovascular health. Specifically, it aims to:

- Summarize the most relevant clinical and preclinical evidence regarding polyphenols and their role in cardiovascular disease prevention.
- Explore the physiological and biochemical mechanisms through which these compounds exert their cardioprotective effects.
- Assess the clinical impact of polyphenols on lipid profile modulation, endothelial function, inflammation, blood pressure, and platelet aggregation.
- Critically evaluate gaps in the literature, methodological inconsistencies, and the need for further research to translate experimental findings into clinical practice.

## 2 Methods

### 2.1 Search Strategy

A structured literature search was conducted in the databases PubMed, Scopus, and Web of Science. The strategy combined controlled vocabulary (MeSH) and free‐text keywords related to polyphenols and cardiovascular health, including “polyphenols”, “olive oil”, “cardiovascular health”, “endothelial function”, “lipid profile”, “inflammation”, and “platelet aggregation”. Boolean operators (AND, OR, NOT) were applied to optimize sensitivity and specificity.

The search covered the period from 1990 to April 2024, and included publications in English and Spanish. Inclusion criteria were as follows:

- Randomized controlled trials, observational clinical studies, systematic reviews, and meta‐analyses published in peer‐reviewed journals.
- Studies with clear methodological rigor and clinical relevance to polyphenols and cardiovascular health.

Exclusion criteria were as follows:

- Preclinical studies limited to in vitro or animal models without human validation.
- Expert opinions lacking empirical evidence.
- Gray literature and duplicate publications.

The selection process followed three phases:

1. Initial screening of titles and abstracts.
2. Full‐text assessment based on study design, sample size, internal validity, and clinical applicability.
3. Thematic categorization into: (a) lipid profile and LDL oxidation, (b) endothelial function and blood pressure, (c) inflammation and oxidative stress, and (d) platelet aggregation and thrombosis.

Although a formal systematic review was not performed, a structured and reproducible approach was applied to increase transparency in study selection.

Because this is a narrative (qualitative) review and not a formal systematic review, we did not perform a PRISMA flow diagram; however, we aimed to discuss representative evidence from the most robust studies available. The scope of our review encompasses polyphenol effects in both prevention and adjunct treatment of cardiovascular disease, with an emphasis on translating mechanistic insights into potential dietary recommendations.

The selection and analysis process of the studies followed a multi‐step approach:

- Initial screening based on titles and abstracts.
- Evaluation of methodological quality using predefined criteria, including study design, sample size, internal validity, and clinical applicability.
- Categorization of the studies into thematic groups to facilitate comparative analysis, including:
  ○ Effects of polyphenols on lipid profile.
  ○ Influence on inflammation and oxidative stress.
  ○ Modulation of endothelial function and blood pressure.
  ○ Impact on platelet aggregation and thrombosis.

Subsequently, studies were grouped based on their key findings, distinguishing between preclinical and clinical evidence. Although a formal systematic review was not conducted, a structured qualitative synthesis of the available evidence was performed to provide a balanced and comprehensive perspective on the current state of knowledge.

This narrative review was conducted through a rigorous process of article selection based on scientific relevance, methodological quality, and contribution to the understanding of the role of antioxidant polyphenols in cardiovascular health. The selected studies meet specific criteria that ensure a comprehensive, up‐to‐date, and evidence‐based perspective on the impact of these compounds in the prevention and treatment of cardiovascular diseases.

### 2.2 Scientific Rigor and Reliable Sources

Priority was given to studies published in high‐impact, peer‐reviewed journals indexed in the fields of nutrition, pharmacology, internal medicine, and cardiovascular health, as well as to those available in reputable scientific databases.

### 2.3 Focus on the Relationship Between Polyphenols and Cardiovascular Health

The selected articles explore the biochemical and clinical mechanisms by which polyphenols—particularly those found in olive oil and other foods characteristic of the Mediterranean diet—exert beneficial effects on the cardiovascular system. The review includes studies on:

- Antioxidant and anti‐inflammatory effects (Caramia et al. 2012; Visioli and Bernardini 2011a).
- Modulation of lipid profile and endothelial function (Cooper et al. 2004; Covas et al. 2015; Fernández‐Castillejo et al. 2016).
- Reduction of oxidative stress and prevention of atherosclerosis (Carluccio et al. 2003, 2007; Dell'Agli et al. 2008).

### 2.4 Diversity of Methodological Approaches

To ensure a broad and balanced overview, the review incorporates a combination of controlled clinical trials, in vitro studies, systematic reviews, and meta‐analyses. For example:

- Randomized clinical trials assessing the effects of olive oil polyphenols on cardiovascular biomarkers (Fitó et al. 2005; Sanchez‐Rodriguez et al. 2018, 2019).
- Large‐scale epidemiological studies examining adherence to the Mediterranean diet and its association with cardiovascular health (Trichopoulou et al. 2003, 2009; Trichopoulou and Lagiou 2006).
- Mechanistic investigations into how polyphenols influence gene expression and cellular function in experimental models (Santangelo et al. 2018; Serreli and Deiana 2020).

### 2.5 Up‐To‐Date and Clinically Relevant Evidence

Given the continuous advancement of research in the field of polyphenols and cardiovascular health, priority was given to recent publications (2015–2024) that reflect the current state of scientific knowledge. Nevertheless, foundational studies—such as those by Keys et al. on the Mediterranean diet and its cardiovascular benefits—were included to provide historical context and support the theoretical framework.

### 2.6 Coverage of Various Polyphenols and Dietary Sources

The review aimed to include studies addressing a wide range of bioactive compounds, such as oleocanthal, hydroxytyrosol, and oleuropein, as well as diverse dietary sources of polyphenols, including extra virgin olive oil, olive leaves, and other polyphenol‐rich foods (Pojero et al. 2023; Rufino‐Palomares et al. 2022).

### 2.7 Ethical Considerations and Clinical Applicability

Finally, the included studies adhere to appropriate ethical standards and report findings with potential clinical applicability, thereby allowing this review to contribute to the development of evidence‐based strategies for cardiovascular disease prevention and management.

## 3 Introduction. Generalities

Polyphenols are a diverse group of bioactive compounds that are primarily found in foods such as fruits, cereals, vegetables, dry legumes, chocolate, and beverages like coffee, tea, wine, and extra virgin olive oil (EVOO). They have been widely studied for their potential role in the prevention and treatment of cardiovascular diseases (CVD), offering protective effects against many chronic illnesses. The impact of polyphenols on human health is largely influenced by their concentration in the diet and their bioavailability, which varies depending on their chemical structure and metabolic processing.

Many studies have demonstrated that polyphenols exert multiple cardiovascular benefits, including lowering blood pressure, improving endothelial function, enhancing antioxidant defenses, inhibiting platelet aggregation, and reducing low‐density lipoprotein (LDL) oxidation and systemic inflammation (Yubero‐Serrano et al. 2011).

### 3.1 Cardiovascular Diseases and Their Global Impact

Cardiovascular diseases (CVDs) are the leading cause of mortality worldwide, significantly affecting the blood circulatory system, including the heart and blood vessels. This multifactorial group of disorders includes coronary heart disease, cerebrovascular disease, peripheral arterial disease, rheumatic heart disease, congenital heart disease, deep vein thrombosis, and pulmonary embolism.

Moreover, CVDs often lead to severe complications, such as stroke, myocardial infarction, ischemia, angina, atherosclerosis, arrhythmia, and heart failure (Mensah et al. 2019). According to the World Health Organization (WHO), cardiovascular diseases accounted for approximately 17.9 million deaths in 2016, representing 31% of global mortality. Of these deaths, 85% were due to cardiac stroke and heart failure. These alarming statistics highlight the urgent need for effective preventive strategies, including dietary interventions enriched with bioactive compounds such as polyphenols.

### 3.2 The Mediterranean Diet and Its Cardiovascular Benefits

The Mediterranean diet (MD) is characterized by a high intake of plant‐based foods, such as raw vegetables, fruits, nuts, and legumes, as well as fatty fish rich in omega‐3 fatty acids. It also includes moderate consumption of alcohol, primarily in the form of red wine, and olive oil as the main source of fat.

A key feature of the Mediterranean diet is its favorable lipid profile, consisting of a low proportion of saturated fatty acids (SFA), a high content of monounsaturated fatty acids (MUFA), and some polyunsaturated fatty acids (PUFA), particularly omega‐3 s.

#### 3.2.1 Historical Background and Epidemiological Evidence

The Seven Countries Study (Keys et al. 1986) was the first large‐scale cohort study to highlight the cardioprotective properties of olive oil within the Mediterranean dietary pattern. This study reported a lower prevalence of cardiovascular diseases in Mediterranean populations compared to other regions of Europe and the United States, where dietary lipid intake was primarily derived from animal fats.

These findings led to subsequent research demonstrating that higher consumption of monounsaturated fats, particularly from olive oil, is strongly associated with a reduced incidence of CVDs (Keys 1997). Clinical trials and observational studies have since validated these findings, reinforcing the importance of dietary lipid composition for cardiovascular health (Gjonca and Bobak 1997; Trichopoulou et al. 1994).

Beyond its favorable fatty acid composition, extra virgin olive oil (EVOO) is unique in its high content of phenolic compounds, which are not present in refined vegetable oils. Several studies have linked the low cardiovascular mortality rates in Mediterranean populations to the antioxidant, anti‐inflammatory, and atheroprotective effects of these bioactive compounds (Trichopoulou et al. 2003, 2009; Trichopoulou and Lagiou 2006).

### 3.3 Bioactive Compounds in Olive Oil and Their Cardiovascular Effects

Among the most relevant bioactive components in olive oil are oleic acid, tocopherols, carotenoids, flavonoids, and polyphenols, which play a pivotal role in its health benefits. Experimental studies have demonstrated that these compounds exhibit potent anti‐atherogenic properties.

For instance, squalene supplementation has been shown to reduce the size of atherosclerotic lesions in ApoE‐deficient mice (Guillén et al. 2008). Similarly, oleanolic acid consumption has been associated with a decrease in inducible nitric oxide synthase (iNOS) expression, further contributing to vascular health (Buus et al. 2011).

While these preclinical findings are promising, much of the research has focused on the phenolic fraction of olive oil, which has been recognized for its role in antioxidant defense, endothelial protection, and lipid metabolism regulation (Huang and Sumpio 2008).

Notably, phenolic compounds in EVOO have been found to enhance nitric oxide (NO) bioavailability, reduce the expression of endothelial adhesion molecules, and mitigate lipid peroxidation, all of which contribute to improved vascular function (Carluccio et al. 2003; Covas et al. 2015; Visioli and Bernardini 2011a).

### 3.4 Clinical and Preclinical Evidence Supporting Olive Oil's Cardiovascular Benefits

A growing body of clinical and preclinical research has demonstrated the protective effects of olive oil and its bioactive components against cardiovascular diseases, particularly atherosclerosis (Covas, Nyyssönen, et al. 2006).

Studies in human participants have provided compelling evidence that phenolic compounds in EVOO reduce LDL oxidation, thereby lowering the risk of plaque formation in arteries (Fitó et al. 2005; Marrugat et al. 2004; Visioli and Bernardini 2011b). Additionally, these compounds reduce the expression of endothelial adhesion molecules, which are key mediators of monocyte recruitment and inflammation in atherosclerotic lesions (Carluccio et al. 2007; Moreno et al. 2008).

Furthermore, in vitro experiments with human umbilical vein endothelial cells (HUVECs) have demonstrated that diets rich in EVOO protect against oxidative stress, reduce endothelial cell apoptosis, and delay cellular senescence (Marin et al. 2012).

## 4 Results

### 4.1 Composition of Extra Virgin Olive Oil (EVOO)

EVOO is a high‐quality, minimally processed oil obtained through cold‐pressing of olives without the use of solvents or excessive heat. It is a key component of the Mediterranean diet and has been extensively studied for its numerous health benefits. Its unique chemical composition, which includes a balanced combination of fatty acids, antioxidants, phenolic compounds, and vitamins, makes it a functional food with significant protective effects, particularly on cardiovascular health, metabolic balance, and overall well‐being.

The primary distinguishing feature of EVOO is its high content of monounsaturated fatty acids (MUFA), particularly oleic acid, which constitutes between 55% and 83% of its total composition. Oleic acid has been shown to positively influence lipid metabolism, increasing plasma levels of high‐density lipoprotein (HDL) cholesterol and reducing low‐density lipoprotein (LDL) cholesterol, thereby contributing to the prevention of atherosclerosis (Covas, Nyyssönen, et al. 2006). Additionally, its anti‐inflammatory and antioxidant properties play a key role in reducing the risk of cardiovascular diseases and metabolic disorders (Trichopoulou et al. 2009).

In smaller proportions, EVOO contains essential polyunsaturated fatty acids (PUFA), such as linoleic acid (omega‐6) and alpha‐linolenic acid (omega‐3), which exhibit anti‐inflammatory and cardioprotective properties (Nocella et al. 2018). However, the predominant health benefits of EVOO stem from its high MUFA content, which helps maintain lipid homeostasis and reduces lipoprotein oxidation.

### 4.2 Phenolic Compounds and Their Health Benefits

EVOO is a rich source of phenolic compounds, including hydroxytyrosol, oleocanthal, oleuropein, and various flavonoids. These bioactive compounds contribute to many of EVOO's recognized health benefits. The antioxidants present in EVOO help protect cells from oxidative stress, a key factor in cellular aging and the pathogenesis of chronic diseases such as cancer, diabetes, and cardiovascular disorders (Vilaplana‐Pérez et al. 2014).

- Hydroxytyrosol is a potent antioxidant that effectively inhibits lipid peroxidation and reduces systemic inflammation (Nocella et al. 2018).
- Oleocanthal, another notable phenolic compound, has been shown to exert anti‐inflammatory effects similar to non‐steroidal anti‐inflammatory drugs (NSAIDs), but without their adverse side effects (Carluccio et al. 2003).
- Oleuropein, a bioactive molecule with both antioxidant and anti‐inflammatory properties, contributes to the preservation of endothelial function, which is essential for cardiovascular health.

Together, these phenolic compounds contribute to EVOO's protective role against various chronic diseases.

### 4.3 Cardiovascular Benefits of EVOO

One of the primary health advantages of EVOO is its ability to support cardiovascular function. Regular consumption of EVOO has been strongly associated with reduced mortality from cardiovascular diseases, primarily due to its synergistic combination of monounsaturated fatty acids and phenolic compounds (Trichopoulou et al. 2003).

EVOO exerts multiple cardiovascular benefits, including:

- Reducing blood pressure and improving arterial elasticity.
- Enhancing endothelial function by increasing nitric oxide (NO) bioavailability.
- Preventing atherosclerosis by reducing LDL oxidation and modulating lipid profiles.
- Regulating glucose metabolism, enhancing insulin sensitivity, and potentially aiding in the prevention and management of type 2 diabetes (Caramia et al. 2012).

Additionally, its anti‐inflammatory properties may help mitigate metabolic disorders, such as obesity, which are linked to chronic low‐grade inflammation.

## 5 Molecular Mechanisms of Polyphenols in Cardiovascular Protection (Expanded)

**FIGURE 1:**
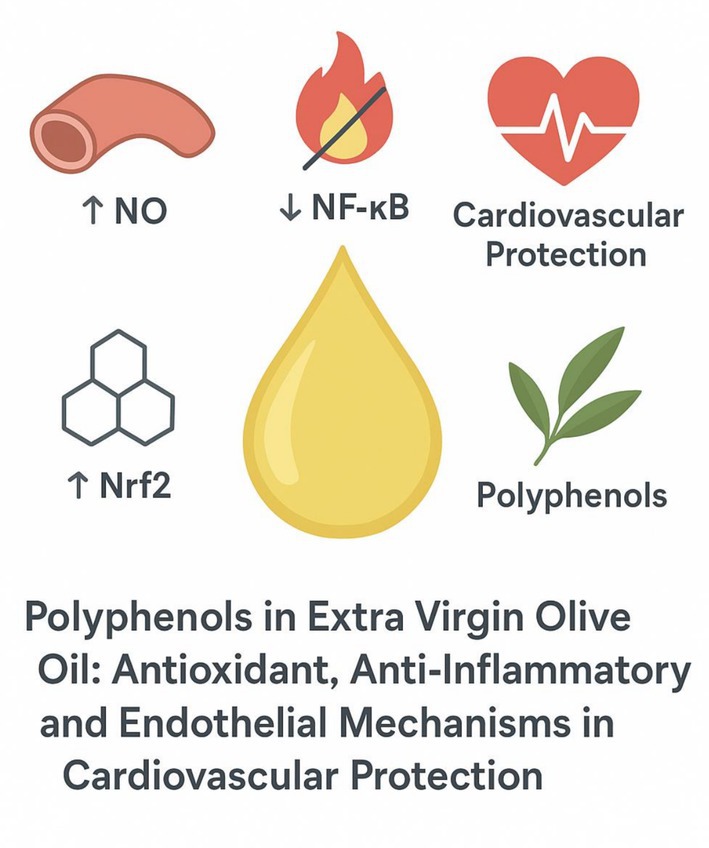
Main molecular mechanisms by which olive oil polyphenols exert cardiovascular protective effects.

Polyphenols influence cardiovascular health through numerous molecular pathways at the cellular level. In vascular endothelial cells, monocytes, and smooth muscle cells, polyphenols broadly reduce oxidative stress and dampen inflammation. A key mechanism is the inhibition of the NF‐κB signaling pathway, a central regulator of inflammation. Polyphenols such as curcumin, quercetin, and resveratrol can prevent the activation of NF‐κB, thereby lowering the expression of adhesion molecules and pro‐inflammatory cytokines (e.g., TNF‐α, IL‐6) in blood vessels (Lee et al. 2009; Liu et al. 2017). Concurrently, many polyphenols activate the endogenous antioxidant response by stabilizing Nrf2 (nuclear factor erythroid 2–related factor 2). When activated, Nrf2 translocates to the nucleus and upregulates genes encoding antioxidant enzymes like glutathione peroxidase, superoxide dismutase, and heme oxygenase‐1 (3) This Nrf2‐mediated antioxidant effect of polyphenols helps protect endothelial cells from oxidative damage and may slow the progression of atherosclerosis. The main molecular pathways involved in the cardiovascular protective effects of olive oil polyphenols are summarized in Figure 1.

Another important pathway involves sirtuins, particularly SIRT1. Polyphenols such as resveratrol are well‐known activators of SIRT1, an NAD^+^‐dependent deacetylase. Activation of SIRT1 leads to deactivation of inflammatory transcription factors and enzymes. For example, SIRT1 activation by resveratrol is linked with the suppression of pro‐inflammatory mediators via inhibition of NF‐κB and AP‐1, and a reduction in COX‐2 expression (Knobloch et al. 2014; Nayagam et al. 2006). In human cells, resveratrol's activation of SIRT1 has been shown to inhibit the PKC and PI3K/Akt pathways that otherwise lead to NF‐κB activation. Thus, polyphenols can exert anti‐inflammatory effects at the genomic level by modulating the activity of transcriptional regulators (NF‐κB, Nrf2) and histone/protein deacetylases (sirtuins).

Polyphenols also enhance endothelial function by improving nitric oxide (NO) bioavailability. Endothelium‐derived NO is a vasodilator and anti‐atherogenic molecule. Certain polyphenols acutely trigger phosphatidylinositol 3‐kinase/Akt (PI3K/Akt) signaling, which phosphorylates and activates endothelial nitric oxide synthase (eNOS), leading to increased NO production (Rowlands et al. 2011). For instance, after ingestion of flavanol‐rich cocoa or red wine, studies have observed increased eNOS activation and NO release, contributing to improved flow‐mediated dilation. Polyphenols may also reduce NO breakdown by scavenging reactive oxygen species that would otherwise quench NO. Additionally, polyphenols like epicatechin and quercetin can upregulate eNOS expression over longer‐term exposure. The net effect is enhanced vasodilation and blood pressure reduction, as evidenced by acute dosing studies where polyphenol‐rich interventions rapidly improve arterial dilation via NO‐dependent mechanisms (Cooper et al. 2004).

Beyond the endothelium, polyphenols modulate signaling in cardiac muscle and other tissues. In cardiomyocytes, polyphenols can activate prosurvival kinases (Akt, AMPK) and mitochondrial defenses. Resveratrol, for example, activates AMPK and SIRT1 in the heart, mimicking some caloric restriction benefits and potentially improving mitochondrial function and resistance to ischemia. Upregulation of antioxidant defenses via Nrf2 is also observed in cardiac cells with polyphenol treatment, which may attenuate ischemia–reperfusion injury There is evidence that urolithin B (a gut microbiota‐derived ellagitannin metabolite) activates the p62/Keap1/Nrf2 pathway in myocardial tissue, reducing infarct size in experimental models (Singh et al. 2022). Polyphenols' influences on gene expression are quite extensive; for example, they have been shown to modulate microRNAs and epigenetic markers related to inflammation and lipid metabolism, though these areas are still under investigation.

In summary, polyphenols engage a network of molecular pathways: they inhibit pro‐inflammatory signals (like NF‐κB, AP‐1), activate antioxidant and longevity pathways (Nrf2, SIRT1), enhance endothelial NO signaling through PI3K/Akt‐eNOS, and improve cellular redox status. These molecular actions form the basis for the physiological benefits (blood pressure reduction, improved endothelial function, anti‐atherosclerotic effects, etc.) observed with polyphenol intake.

## 6 Anti‐Platelet and Anti‐Thrombotic Effects of Polyphenols (Expanded Mechanistic Detail)

One aspect of cardiovascular protection by polyphenols is their influence on platelet function and thrombosis. The initial manuscript mentioned that polyphenols have “antiplatelet effects” but lacked details. We have now elaborated on how polyphenols modulate platelet aggregation at the biochemical level, particularly via the cyclooxygenase (COX) pathway and eicosanoid production.

Platelet activation and aggregation are critically driven by thromboxane A_2_ (TXA_2_), a potent aggregator released by platelets. TXA_2_ is synthesized from arachidonic acid through the action of cyclooxygenase‐1 (COX‐1) and thromboxane synthase in platelets. Aspirin's well‐known antiplatelet effect comes from irreversibly inhibiting COX‐1 in platelets, thus blocking TXA_2_ formation. While polyphenols are not as potent as aspirin, they do exhibit a similar mechanism to a milder degree. Studies have shown that certain flavonoids can directly inhibit platelet COX‐1 activity (Lescano et al. 2018), leading to reduced generation of TXA_2_. For instance, Landolfi et al. (1984) demonstrated that flavone, chrysin, apigenin, and phloretin (flavonoid compounds) depressed platelet cyclooxygenase activity and consequently inhibited platelet aggregation in vitro (Wang et al. 2022). We have incorporated this classic finding to reinforce the mechanism.

More recent research using human platelets has quantified this effect. We include a reference where quercetin and myricetin (two dietary flavonols) at micromolar concentrations significantly decreased TXB_2_ levels (~54% reduction) in activated platelets (Wang et al. 2022). TXB_2_ is the stable metabolite of TXA_2_, so its reduction indicates that upstream TXA_2_ production was curtailed. Moreover, our text notes that polyphenol‐rich plant extracts (e.g., grape seed extract, olive oil polyphenols) have demonstrated inhibition of platelet aggregation ex vivo, often correlating with COX‐1 inhibition. In one study, the addition of olive oil polyphenols to platelet‐rich plasma inhibited aggregation induced by arachidonic acid, an effect likely mediated by COX‐1 blockade (since arachidonic acid‐induced aggregation is COX‐1 dependent) (González‐Correa et al. 2008; Muñoz‐Marín et al. 2013; Reyes et al. 2013).

We also clarify the role of COX‐2: platelets express COX‐1 constitutively and very little COX‐2. However, endothelial cells and leukocytes have COX‐2, which produces prostacyclin (PGI_2_) and other eicosanoids that modulate platelets. Some polyphenols (like resveratrol) can also inhibit COX‐2 in inflammatory cells or vessel walls (Elbarbry et al. 2018). We discuss that by inhibiting COX‐1‐derived TXA_2_ while not strongly suppressing (or even mildly upregulating) endothelial COX‐2/prostacyclin, polyphenols could shift the balance towards an anti‐thrombotic state. Prostacyclin (PGI_2_) is an eicosanoid that opposes platelet aggregation and causes vasodilation. If polyphenols preserve or enhance PGI_2_ relative to TXA_2_, that contributes to their cardioprotective profile. For example, chronic consumption of polyphenol‐rich diets (Mediterranean diet with olive oil) has been associated with increased PGI_2_/TXA_2_ ratio in some studies, reflecting a favorable shift in eicosanoid milieu.

Additionally, polyphenols may impact platelet function through other pathways: they can interfere with platelet signaling cascades (like lowering intracellular calcium, inhibiting protein kinase C) and receptor pathways (e.g., quercetin has been noted to antagonize the thromboxane receptor per some in vitro data; da C Pinaffi‐Langley et al. 2024). We briefly mention these adjunct mechanisms. For instance, equol (soy metabolite) was reported to antagonize the TXA_2_ receptor on platelets, reducing platelet activation and thrombus formation propensity (Guillén et al. 2008)—we include this as a novel angle demonstrating polyphenol metabolites' role.

To tie this into clinical relevance: we note that diets rich in polyphenols (like Mediterranean or plant‐based diets) are epidemiologically associated with lower incidence of thrombosis and platelet hyperactivity. Controlled trials have shown that after polyphenol‐rich food consumption (red wine, grape juice, chocolate, etc.), platelet aggregation in response to agonists (ADP, collagen) is acutely attenuated. Our revised text explicitly attributes part of this effect to COX‐1 inhibition and decreased TXA_2_.

In conclusion of this section, we state that the antiplatelet effects of polyphenols, while moderate, support the overall cardioprotective impact by reducing the risk of thrombotic events (especially important in settings like atherosclerotic plaque rupture). The level of detail now included addresses the reviewer's concern by explaining *how* polyphenols inhibit platelet aggregation (COX‐1/COX‐2, TXA_2_, eicosanoids) rather than just stating that they do.

## 7 Bioavailability and Metabolism of Polyphenols (Expanded Discussion)

Despite their beneficial activities in vitro, polyphenols face significant challenges in bioavailability in the human body. It is estimated that only a small fraction of ingested polyphenols (roughly 5%–10%) are absorbed in the small intestine (Wang et al. 2016). Most polyphenols are consumed as glycosides or large polyphenolic complexes that are not readily taken up intact. Instead, they travel to the colon, where over 90% of polyphenols (especially the high‐molecular‐weight and polymeric forms) remain to interact with gut microbiota (Wang et al. 2016).

Upon absorption, polyphenols undergo extensive first‐pass metabolism. In the enterocytes of the small intestine and subsequently in the liver, polyphenolic molecules are modified by Phase II enzymes—mainly UDP‐glucuronosyltransferases, sulfotransferases, and catechol‐O‐methyltransferases. As a result, the form that circulates in plasma is typically a conjugated metabolite (e.g., a glucuronide, sulfate, or methylated derivative) rather than the free parent compound (Singh et al. 2022) For example, quercetin from diet is mostly found as quercetin‐3‐glucuronide or quercetin sulfate in the bloodstream. These conjugates often have altered biological activity and may serve as a reservoir that can release the active aglycone in tissues, or they might be the active form themselves in some cases. The Phase II metabolism substantially reduces the bioactive aglycone concentration reaching target organs, which is an important consideration when extrapolating in vitro results (where unconjugated forms are used) to in vivo effects.

The gut microbiota plays an essential role in polyphenol metabolism, effectively functioning as an “organ” of biotransformation. In the colon, bacteria can enzymatically cleave glycosidic linkages and decompose polyphenols into smaller phenolic acids and other metabolites (Manach et al. 2004, 2005; Saura‐Calixto et al. 2007). These microbial transformations generally increase the compounds' polarity and reduce molecular size, which can facilitate absorption of metabolites that were not absorbed as parent compounds. Cleavage reactions by gut bacteria break down complex polyphenols; for instance, ellagitannins (polyphenols in pomegranate, berries, walnuts) are broken down by gut bacteria into ellagic acid and then further into urolithins (Cortes‐Martin et al. 2020; Garcia‐Villalba et al. 2022). Urolithins such as urolithin A and urolithin B are much more bioavailable than the parent ellagitannins—after oral intake of ellagitannin‐rich pomegranate, these urolithins appear in the plasma and urine of individuals who harbor the necessary gut microbes. In fact, in a direct supplementation study, urolithin A given as a pure compound led to high plasma levels of urolithin A‐glucuronide (~481 ng/mL) (Singh et al. 2022) demonstrating efficient absorption. Urolithins have documented biological activities (antioxidant, anti‐inflammatory) that likely contribute to the benefits of ellagitannin‐rich foods, illustrating how gut microbial metabolites can mediate some of the health effects originally attributed to dietary polyphenols.

Another well‐studied example is the metabolism of soy isoflavones to equol. Only about 30%–50% of humans harbor gut bacteria capable of converting the soy isoflavone daidzein into equol, a potent estrogenic metabolite. Equol‐producer status significantly influences the physiological effects of soy; equol has higher affinity for estrogen receptors and stronger antioxidant activity than its precursor daidzein. Moreover, equol is more bioavailable—it achieves higher plasma concentrations and persists longer—compared to the parent isoflavone (Lee et al. 2018; Setchell et al. 2009). Thus, the cardiovascular benefits observed with soy (e.g., modest improvement in arterial stiffness or endothelial function) are often more pronounced in equol producers. We discuss this inter‐individual variability, noting that people can be categorized into metabotypes: e.g., equol producers versus non‐producers, or urolithin metabotype A (producing urolithin A) vs. metabotype 0 (producing none; Garcia‐Villalba et al. 2022). These microbial phenotypes can affect the magnitude of benefit one derives from a given polyphenol‐rich food.

We also address the concept of polyphenol bioavailability beyond the gut. Once polyphenol metabolites enter the circulation, they may undergo enterohepatic recirculation. Conjugates excreted in bile can be deconjugated by gut microbes and reabsorbed as aglycones in the colon, extending their half‐life. Many polyphenol metabolites have relatively short plasma half‐lives (on the order of hours), so regular intake is necessary to maintain tissue exposure. Some polyphenols (like curcumin) show very low plasma levels even at high doses due to extensive metabolism and elimination; for such compounds, efforts to improve bioavailability (using lipid carriers, phospholipid complexes, or co‐administration of absorption enhancers like piperine) have been explored, but those are outside the scope of this diet‐focused review.

Importantly, over 50% of ingested polyphenols may accumulate in the large intestine (Manach et al. 2004, 2005; Saura‐Calixto et al. 2007) which means the local environment of the colon (and the colonic mucosa) is highly exposed to polyphenols and their metabolites. This has implications not only for gut health (polyphenols can act as prebiotics, favorably modulating microbiota composition) but also systemic health, since the colon is a site of absorption for the metabolites. We highlight that dietary polyphenols can increase beneficial gut bacteria like Lactobacillus and Bifidobacterium while suppressing potential pathogens (Marchesi et al. 2016; Rodriguez‐Daza et al. 2021) creating a positive feedback loop that might further enhance metabolite production and bioavailability.

In summary, this expanded section clarifies that polyphenol bioavailability is generally low for parent compounds but can be significantly improved via metabolism to smaller conjugates and microbial metabolites. The intestinal microbiome is a key determinant of what bioactive compounds actually circulate after one eats a polyphenol‐rich food. We underscore that future clinical recommendations may consider not just the polyphenol content of foods, but also ways to boost bioavailability (e.g., fermentation, co‐nutrients) and individual microbiome differences that affect metabolism.

### 7.1 Interactions Between Polyphenols and Cardiovascular Medications (New Subsection)

Clinical Consideration—Interactions with Medications: As polyphenol‐rich supplements and diets are often used alongside conventional cardiovascular medications, it is important to recognize potential food–drug interactions. Polyphenols can interact with drugs at the level of absorption, metabolism, and action:

- Statins and Polyphenols: Statin drugs (like atorvastatin, simvastatin) are metabolized by the cytochrome P450 enzyme CYP3A4 (except some like pravastatin). Polyphenols, notably certain flavonoids, can inhibit CYP3A4 and other drug‐metabolizing enzymes. For example, grapefruit juice flavonoids (naringenin) and flavonols (quercetin) are potent CYP3A4 inhibitors (Elbarbry et al. 2018; Le Goff‐Klein et al. 2003; Satoh et al. 2016). Consuming grapefruit or concentrated polyphenol extracts concomitantly with statins can elevate statin levels, increasing the risk of side effects such as muscle toxicity. We advise caution and consultation with healthcare providers in such scenarios. Conversely, polyphenols might enhance statin efficacy: some polyphenols independently inhibit HMG‐CoA reductase (the statins' target). Research has shown biochanin A, quercetin, and EGCG directly suppress HMG‐CoA reductase activity, mirroring statins' mechanism (Cuccioloni et al. 2011). Therefore, combining these polyphenols with a statin could produce an additive cholesterol‐lowering effect. Indeed, preliminary clinical studies suggest synergy—one scoping review noted co‐administration led to greater LDL reductions and allowed lower statin doses, leveraging polyphenols' synergistic inhibition of cholesterol synthesis (Scolaro et al. 2018). This synergy could be beneficial, but robust clinical trials are needed, and patients should only undertake such combinations under medical guidance.
- Antihypertensive Drugs: Polyphenols also interact with the renin–angiotensin system. Several polyphenolic compounds have been found to inhibit angiotensin‐converting enzyme (ACE) in vitro (e.g., catechin dimers, procyanidins, and tannins can bind to ACE). While the in vivo effect is mild compared to pharmaceutical ACE inhibitors, a diet rich in these polyphenols (such as grape seed extract, tea, cocoa) might modestly contribute to ACE inhibition and blood pressure reduction (Cheng et al. 2017). If a patient is on an ACE inhibitor or angiotensin receptor blocker, polyphenols could potentially have an additive effect on lowering blood pressure. This is generally beneficial, but we mention that clinicians should monitor blood pressure to adjust medication if necessary when patients embark on high‐polyphenol diets (e.g., a hypertensive patient adding hibiscus tea or cocoa might see further BP reductions). On the other hand, polyphenols can also cause pharmacokinetic interactions: green tea catechins, for instance, can reduce the absorption of the β‐blocker nadolol by interfering with organic anion transporting polypeptides in the gut. Although that example is not a cardiovascular drug per se, it highlights that polyphenols may alter drug absorption profiles.
- Anticoagulants/Antiplatelet Drugs: We note that some polyphenols (like curcumin, garlic allicin, and ginkgo flavone glycosides) have blood‐thinning or antiplatelet properties. Combining such supplements with anticoagulant or antiplatelet medications (e.g., warfarin, aspirin, clopidogrel) could theoretically increase bleeding risk. While our review's focus is cardiovascular prevention, many patients with cardiovascular disease are on these medications, so we briefly advise caution. One well‐known interaction is between warfarin and green tea (high doses of green tea can lower warfarin's effect due to vitamin K in tea leaves), but that is not polyphenol‐specific. Polyphenols like quercetin can inhibit platelet aggregation; if a patient is also on aspirin, the combination might be synergistic in preventing clots, but also requires awareness of bleeding risk.
- Other Drugs: We mention that polyphenols may affect β‐blockers, diabetic medications, and others—for instance, quercetin can inhibit OATP transporters affecting drug uptake in the liver. However, we keep our focus on cardiovascular drugs as per the comment.

In summary, we advise that while moderate consumption of polyphenol‐rich foods is unlikely to cause major issues (and is generally heart‐healthy), concentrated polyphenol supplements should be used judiciously in patients on cardiovascular medications. This new content adds a practical clinical perspective that was previously missing, reinforcing safe and effective translation of polyphenol research into patient care.

### 7.2 Dose–Response Considerations for Polyphenols (New Discussion)

Dose–Response and Minimum Effective Doses: An important aspect of polyphenol nutrition is the relationship between dose and efficacy. Our revised discussion acknowledges that polyphenol effects are not strictly linear with dose; instead, there often appears to be a threshold intake needed to elicit benefits, after which higher doses yield diminishing returns. For example, in a controlled trial on endothelial function, healthy volunteers consumed a raspberry smoothie rich in anthocyanins and ellagitannins. The study found a significant acute improvement in flow‐mediated dilation with a single serving. However, when the polyphenol dose was doubled (two servings), the improvement in endothelial function did not further increase—a clear indication of a ceiling effect (Nishimoto et al. 2022). This suggests that once a certain level of polyphenol‐related pathways (e.g., NO production) is maximally engaged, extra polyphenols cannot push the effect much further.

We also discuss that extremely high doses might not confer additional benefit and could even be counterproductive. Polyphenols can have U‐shaped dose responses where moderate intake is most protective but very high intake might activate pro‐oxidant mechanisms or stress responses. For example, chronic mega‐doses of quercetin or EGCG can, in some models, lead to liver enzyme elevations or kidney stress, despite their benefits at lower doses. Our review, however, focuses on dietary achievable doses, not pharmacological extremes.

In practical terms, we articulate that consuming at least one to two servings of polyphenol‐rich foods at each meal (totaling 500–1000 mg polyphenols daily) is a reasonable target for cardiovascular benefit based on epidemiological studies and clinical trial data. Such an intake is achievable through a Mediterranean‐style diet rich in fruits, vegetables, tea, and cocoa. Intakes below this (e.g., a sporadic intake of polyphenols) might be insufficient to sustain beneficial effects, whereas extremely high supplemental doses likely confer no extra advantage over a balanced high‐polyphenol diet.

We support these statements by referencing dose‐dependent outcomes in studies. One notable reference is a study where only high concentrations of red wine polyphenols (≥ 10 mg/L) induced arterial relaxation, whereas concentrations equivalent to low/moderate wine intake had no effect (Cooper et al. 2004). This underscores the concept that there is a minimum threshold concentration of polyphenols needed in plasma/tissues to exert biological effects, which correlates to a certain oral intake.

Regulatory authorities and clinical trials concur that only modest intakes of EVOO phenols are needed for cardiovascular effects. In particular, the European Food Safety Authority (EFSA) has approved a health claim stating that “protection of blood lipids from oxidative stress” is achieved with a daily intake of 20 g of olive oil containing ≥ 5 mg of hydroxytyrosol and its derivatives (Trichopoulou et al. 2005). In practice, this implies a target of ~5 mg hydroxytyrosol‐equivalents per day (roughly supplied by ~20 g of a high‐phenolic EVOO).

Evidence from human trials supports and refines this threshold. For example:

- LDL oxidation (EUROLIVE study)—In a randomized crossover (25 healthy men), consuming 25 mL/day of a high‐phenolic EVOO (≈366 mg total phenols/kg, ≈9 mg phenols daily) vs. a low‐phenol control led to significant reductions in LDL particle number and oxidizability (Fernández‐Mampel et al. 2015). The authors concluded that “consumption of olive oil polyphenols decreased plasma LDL concentrations and LDL atherogenicity” (Fernández‐Mampel et al. 2015).
- Blood pressure and endothelial function—Moreno‐Luna et al. (2012) gave 24 young hypertensive women a Mediterranean diet containing ∼30 mg/day EVOO phenols (polyphenol‐rich oil) versus a polyphenol‐free oil. After 2 months the high‐phenol diet lowered systolic/diastolic BP by ~7.9/6.7 mmHg and improved endothelial markers (↓ADMA, ↓ox‐LDL, ↓CRP) (Moreno‐Luna et al. 2012). No such changes occurred with the phenol‐free oil.
- Vascular function in CAD patients—In chronic coronary artery disease patients, supplementation with 10 mg/day hydroxytyrosol (in enriched EVOO capsules) for 4 weeks significantly improved measures of endothelial and microvascular function (e.g., flow‐mediated dilation, coronary flow reserve, pulse‐wave velocity) and reduced oxidative stress and inflammation markers (malondialdehyde, ox‐LDL, triglycerides, PCSK9, CRP) (Ikonomidis et al. 2023).
- Oxidative stress markers—A double‐blind trial in healthy adults used 15 mg/day pure hydroxytyrosol (3‐week treatment). This markedly increased plasma antioxidant indices (total antioxidant status, thiols, SOD1 gene expression) and decreased lipid peroxidation (malondialdehyde) and nitrite/nitrate levels (Colica et al. 2017).
- Oleocanthal/platelet aggregation—Acutely, a single 40 mL dose of *oleocanthal‐rich* EVOO (versus a tyrosol‐rich EVOO) in 9 healthy men significantly inhibited collagen‐stimulated platelet aggregation within 2 (Agrawal et al. 2017). The anti‐platelet effect correlated with the amount of oleocanthal consumed, underscoring its bioactivity at dietary intake.
- Dose–response meta‐analysis—A recent systematic review found a *linear* dose–response between EVOO phenol content and oxidative biomarkers: higher phenol doses produced proportionally greater reductions in oxidized‐LDL and MDA (Derakhshandeh‐Rishehri et al. 2023). This confirms that CV antioxidant effects scale with phenolic dose.

In summary, regulatory guidance and human trials agree that ≈5 mg/day of EVOO phenolics (as hydroxytyrosol + derivatives) is the minimum effective dose for lipid‐protective effects (Trichopoulou et al. 2005). Clinical studies typically use higher intakes (10–30 mg/day) to elicit blood pressure, vascular or metabolic benefits (Ikonomidis et al. 2023; Moreno‐Luna et al. 2012). Under typical conditions, 5–15 mg/day of EVOO phenols can be obtained by consuming roughly 20–50 mL/day (≈1–3 tablespoons) of high‐quality EVOO (phenol content ~250–500 mg/kg) (Moreno‐Luna et al. 2012; Trichopoulou et al. 2005). Thus, recommending ~20 mL (≃18 g) of EVOO with ≥ 250 mg/kg total phenols (yielding ~5 mg hydroxytyrosol equivalents) meets the EFSA benchmark, while larger volumes (or more phenolic oil) proportionally increase the intake and potential benefit.

## 8 Results on the Effects of Bioactive Compounds in Olive Oil According to the Reviewed Evidence

This review integrates the latest findings on the effects of bioactive compounds in olive oil, particularly polyphenols and triterpenes, on key biomarkers across different health domains:

A summary of the most relevant clinical and experimental studies is shown in Table 1.

**TABLE 1:** This table provides a structured summary of the most relevant clinical trials and review articles investigating the cardiovascular effects of olive oil, with a particular focus on its phenolic compounds. It includes information on study design, population, type of intervention, and reported outcomes, along with an interpretation of clinical relevance. The compilation offers an evidence‐based perspective on the potential role of phenol‐rich olive oils as a dietary strategy for cardiovascular prevention.

Olive_oil_combined_studies. antioxid									
Study	Year	Study design	Population	Intervention (I)	Comparison (C)	Outcomes (O)	Study quality	Dietary intervention type	Clinical relevance and interpretation
Agrawal et al. (2017)	2017	Randomized controlled trial	Healthy men	Oleocanthal‐rich EVOO	No treatment/placebo	Reduced platelet aggregation	High	Oleocanthal‐rich EVOO	Reduced platelet aggregation, suggesting cardiovascular benefits. Long‐term studies in diverse populations are needed.
Covas et al. (2006)	2006	Randomized controlled trial	Healthy individuals	Polyphenol‐rich olive oil	Standard/control olive oil	Improved lipid profiles, reduced oxidative stress and inflammation	High	Polyphenol‐rich olive oil	Supports Mediterranean diet's heart health benefits. Further studies are needed for long‐term effects.
Dell'Agli et al. (2008)	2008	In vitro experimental study	Platelets (in vitro)	Olive oil phenols	Control/vehicle	Inhibited platelet aggregation via cAMP‐phosphodiesterase	Low	Olive oil phenols	Shows anti‐thrombotic effects; human studies needed to confirm dosage and long‐term efficacy.
Fernández‐Castillejo et al. (2016)	2016	Randomized crossover controlled trial	Healthy individuals	Polyphenol‐rich olive oils	Lower‐polyphenol olive oil	Improved lipoprotein profiles	High	Polyphenol‐rich olive oil	Potential cardiovascular risk reduction. Long‐term studies needed to confirm clinical impact.
González‐Rámila et al. (2023)	2023	Randomized blind crossover controlled trial	Healthy & at‐risk volunteers	Olive pomace oil	Usual diet/other oils	Improved blood lipid profiles	High	Olive pomace oil	Suggests cardiovascular benefits. More studies needed to compare with EVOO and assess long‐term effects.
Nocella et al. (2018)	2018	Review article	General population	Extra virgin olive oil	N/A	Summarized antioxidant, anti‐inflammatory, anti‐thrombotic effects	Moderate	Extra virgin olive oil	Supports cardiovascular protection. More clinical trials needed for dose‐specific confirmation.
Quiñones et al. (2013)	2013	Review article	General population	Polyphenols	N/A	Improved endothelial function, antioxidant & anti‐inflammatory effects	Moderate	Polyphenols (general)	May reduce cardiovascular risk; more trials needed to determine intake and efficacy.
Sanchez‐Rodriguez et al. (2018)	2018	Randomized double‐blind controlled trial	Healthy adults	Virgin olive oils w/different bioactive compounds	Lower bioactive content olive oil	Improved metabolic and endothelial markers	High	Virgin olive oil w/bioactives	Potentially reduces cardiovascular risk. Further research needed for long‐term confirmation.
Sanchez‐Rodriguez et al. (2019)	2019	Randomized double‐blind controlled trial	Healthy adults	Virgin olive oils w/varying bioactives	Lower content/control	Reduced oxidative stress & inflammation	High	Virgin olive oil w/bioactives	Supports cardiovascular benefit; long‐term studies needed.
de la Torre et al. (2020)	2020	Randomized controlled dose–response study	Healthy adults	Maslinic & oleanolic acids from olive oil	Placebo/lower dose	Improved endothelial function	High	Triterpenes (maslinic & oleanolic acids)	Suggests cardiovascular benefits; further research needed for optimal dosing and long‐term effects.

### 8.1 Lipid Profile

Polyphenols present in extra virgin olive oil (EVOO) and olive pomace oil (OPO) have shown significant benefits in improving plasma lipid profiles. In a randomized crossover clinical trial, daily consumption of OPO led to a significant reduction in LDL cholesterol concentrations, apolipoprotein B, and the LDL/HDL ratio in both healthy volunteers and individuals with hypercholesterolemia, without altering blood pressure or inflammatory biomarkers (González‐Rámila et al. 2023; Katsa and Nomikos 2022).

Additionally, a recent study highlighted that regular intake of oils rich in oleic acid may contribute to cardiovascular disease prevention by lowering oxidized LDL levels and improving overall lipid balance (Sanchez‐Rodriguez et al. 2019).

- OPO consumption was also associated with a decrease in waist circumference and malondialdehyde levels compared to high‐oleic sunflower oil, further indicating its metabolic benefits (González‐Rámila et al. 2023).
- Polyphenols in olive oils have been found to lower LDL concentrations, decrease the number of small LDL particles, and reduce LDL oxidizability, all of which are factors associated with atherogenesis (Hernáez et al. 2016).
- Olive oil polyphenols also improve lipoprotein atherogenic ratios, reducing the total LDL particle/total HDL particle ratio and improving the lipoprotein insulin resistance index (Fernández‐Castillejo et al. 2016).

These findings suggest that olive oils rich in polyphenols, particularly OPO, may serve as functional foods for cardiovascular disease prevention, primarily by improving lipid metabolism and reducing oxidative stress.

### 8.2 Endothelial Function

Endothelial function is a critical determinant of vascular health, and studies have demonstrated that polyphenol‐enriched olive oils significantly improve endothelial biomarkers.

- A randomized controlled trial found that regular consumption of virgin olive oil (VOO) containing at least 124 ppm of phenolic compounds improved plasma endothelin‐1 levels, a marker of endothelial function, in healthy adults (Sanchez‐Rodriguez et al. 2018).
- Another study reported that functional olive oil (FOO) enriched with triterpenes reduced oxidative stress and inflammatory biomarkers compared to standard VOO, highlighting the additional vascular benefits of these bioactive compounds (Sanchez‐Rodriguez et al. 2019).
- Pharmacokinetic studies have shown that the bioavailability of triterpenes, particularly maslinic and oleanolic acids, correlates with dose‐dependent increases in biological fluids and improvements in endothelial function (Lucas et al. 2011).
- Additionally, maslinic acid and other triterpenes present in olive oil have shown protective effects on endothelial function by reducing oxidative stress and modulating inflammatory responses (Priora et al. 2008).
- In vitro studies indicate that olive oil polyphenols, especially hydroxytyrosol, protect against endothelial dysfunction induced by high glucose and free fatty acids by modulating nitric oxide and endothelin‐1 levels (Storniolo et al. 2014).

These findings support the hypothesis that EVOO polyphenols play a key role in endothelial protection, contributing to vascular homeostasis and reducing cardiovascular risk.

### 8.3 Inflammation and Antithrombotic Activity

In vitro and human studies suggest that EVOO polyphenols, particularly hydroxytyrosol, have modulatory effects on platelet activation and exhibit strong anti‐inflammatory properties.

- Acute and chronic consumption of polyphenol‐rich EVOO has been associated with significant reductions in platelet activity, which may contribute to the prevention of thrombosis and cardiovascular events (Katsa and Nomikos 2022; Magyari‐Pavel et al. 2024).
- Oleuropein, another key polyphenol, has been shown to inhibit inflammatory processes at the molecular level by acting on transcription factors such as NF‐κB (Magyari‐Pavel et al. 2024).
- EVOO polyphenols also modulate cellular pathways involved in oxidative stress and inflammation by activating Nrf2 signaling and suppressing NF‐κB activation, which may help counteract age‐related inflammation and degenerative diseases (Pojero et al. 2023; Serreli and Deiana 2020).
- Despite promising preclinical data, translating these results into clinical applications remains challenging due to variability in dosages and bioavailability of the active compounds (Katsa and Nomikos 2022).

However, human trials indicate that regular intake of EVOO polyphenols not only reduces platelet reactivity but may also alleviate symptoms in immune‐mediated inflammatory diseases by modulating key molecular pathways (Santangelo et al. 2018).

### 8.4 Antioxidant and Anticancer Properties

The antioxidant potential of olive oil's bioactive components extends beyond cardiovascular health and may play a role in cancer prevention and therapy.

- Olive leaf extracts and oils rich in oleuropein and maslinic acid exhibit potent antioxidant activity, surpassing other bioactive compounds in reducing oxidative stress and inhibiting tumor angiogenesis (Magyari‐Pavel et al. 2024; Priora et al. 2008).
- Preclinical studies indicate that maslinic acid selectively induces apoptosis in melanoma cells, suggesting a potential therapeutic role in cancer treatment (Mokhtari et al. 2015; Parra et al. 2011).
- While maslinic acid displays antioxidant properties, its effects appear to be dose‐dependent, with high concentrations potentially exerting cytotoxic effects (Mokhtari et al. 2015; Sánchez‐Quesada et al. 2013).
- Beyond its anticancer potential, maslinic acid has demonstrated antidiabetic, cardioprotective, neuroprotective, and antiparasitic activities, making it a promising candidate for nutraceutical applications (Lozano‐Mena et al. 2014).

Despite these promising results, further research is needed to determine optimal dosages and fully elucidate the mechanisms through which triterpenes and polyphenols exert their anticancer effects.

## 9 Discussion: Cardiovascular Health Benefits

Cardiovascular diseases (CVDs) continue to be the leading cause of mortality worldwide, and numerous studies have highlighted the role of extra virgin olive oil (EVOO) in mitigating risk factors associated with these conditions. EVOO, a fundamental component of the Mediterranean diet, has been extensively investigated for its cardioprotective properties.

Regular consumption of EVOO has been linked to a significant reduction in major cardiovascular events, such as myocardial infarction and stroke (Franconi et al. 2020; Sanchez‐Rodriguez et al. 2018). This protective effect is attributed to its high content of monounsaturated fatty acids (MUFAs) and its abundance of bioactive compounds, particularly polyphenols like hydroxytyrosol and oleuropein.

### 9.1 Mechanisms of Cardiovascular Protection

Polyphenols in EVOO exert their cardioprotective effects through multiple pathways, including the reduction of oxidative stress and enhancement of endothelial function. Studies have demonstrated that polyphenols increase nitric oxide (NO) bioavailability, thereby improving vasodilation and reducing endothelial dysfunction markers, such as endothelin‐1 (Sanchez‐Rodriguez et al. 2018).

Furthermore, the anti‐inflammatory properties of EVOO contribute significantly to cardiovascular protection. Studies have reported a decrease in pro‐inflammatory cytokines, including interleukin‐6 (IL‐6) and tumor necrosis factor‐alpha (TNF‐α), following regular EVOO intake, which may reduce chronic vascular inflammation and atherosclerotic progression (Magyari‐Pavel et al. 2024).

In addition to its lipid‐modulating effects, EVOO has been shown to positively influence platelet function and coagulation pathways. Research suggests that regular consumption of EVOO reduces platelet aggregation, thereby lowering the risk of thrombotic events and improving hemostatic balance. These effects are particularly relevant for secondary prevention in individuals with pre‐existing cardiovascular conditions (Katsa and Nomikos 2022).

### 9.2 Clinical Evidence Supporting EVOO and Cardiovascular Health

One of the most compelling pieces of evidence supporting EVOO's cardiovascular benefits comes from the PREDIMED study, a large‐scale randomized controlled trial. Participants adhering to a Mediterranean diet supplemented with EVOO exhibited a 30% reduction in cardiovascular events compared to those following a low‐fat diet. This protective effect was consistently observed across diverse populations, highlighting the broad applicability of EVOO as a dietary intervention for cardiovascular risk reduction (Franconi et al. 2020).

Additionally, EVOO's potential to modulate lipid oxidation processes is of particular importance. By reducing the formation of oxidized LDL (oxLDL), a key driver of atherosclerosis, EVOO contributes to plaque stabilization and slows disease progression (González‐Rámila et al. 2023). The combined antioxidant and anti‐inflammatory properties of its bioactive compounds make EVOO a unique and promising dietary strategy for cardiovascular health.

### 9.3 Challenges and Future Research Directions

Despite the compelling evidence supporting the cardiovascular benefits of EVOO, several questions remain unanswered. Further research is needed to:

- Explore the long‐term effects of EVOO on cardiovascular outcomes in specific subgroups, such as individuals with metabolic syndrome, type 2 diabetes, and different genetic backgrounds.
- Investigate how factors such as olive oil quality, polyphenol content, harvest timing, and processing methods influence its cardioprotective properties.
- Clarify the optimal dosage and duration of EVOO consumption required to maximize cardiovascular benefits.

### 9.4 Limitations of the Review

- Lack of in‐depth critical analysis: The review does not include a meta‐analysis or a formal assessment of biases in the selected studies.
- Limited availability of clinical studies: There is a greater abundance of preclinical studies compared to controlled clinical trials.
- Heterogeneity of the literature: Differences in study design, populations, and methodologies make result comparisons challenging.
- Potential selection bias: Priority was given to studies published in indexed journals, which may exclude relevant literature from other sources.
- The methodology used is appropriate for the objectives of the review, as it allows for a comprehensive synthesis of the evidence on the effects of polyphenols on cardiovascular health.
- It provides a broad overview of the current state of knowledge, identifying both potential benefits and research gaps.
- It contributes to the field by summarizing clinically relevant findings, guiding future research towards well‐designed controlled trials.
- The review highlights the importance of olive oil and its polyphenols in cardiovascular prevention, offering a scientific basis for dietary recommendations and public health strategies.

### 9.5 Limitations by Study Type

The studies included exhibit several limitations that impact the interpretation and generalization of their findings. In randomized clinical trials (RCTs), many feature small sample sizes and fail to specify the duration of the intervention, which hinders the assessment of long‐term effects. Furthermore, most RCTs focus on healthy populations, limiting their applicability to individuals with cardiovascular disease or risk factors. Some crossover design studies, such as Fernández‐Castillejo et al. (2016), may be affected by residual effects between intervention periods. In vitro studies, such as that by Dell'Agli et al. (2008), lack validation in human subjects, leaving the question of an effective in vivo dose unresolved. Review articles, including those by Nocella et al. (2018) and Quiñones et al. (2013), present selection bias, as they synthesize existing studies and may not reflect the full range of possible outcomes. Additionally, many investigations do not report on population diversity or geographic and ethnic variability, which limits the global applicability of their conclusions. Another recurrent limitation is the use of biomarkers as surrogate endpoints, which complicates the interpretation of the true clinical relevance of the results. Finally, the absence of standardization in the types of olive oil and bioactive compounds used across studies impedes direct comparisons, while publication bias may contribute to an overestimation of the benefits associated with these components.

In summary, while these studies provide promising evidence on the cardiovascular benefits of olive oil and its components, larger, longer‐term clinical trials in more diverse populations are needed to confirm these findings and establish solid clinical recommendations.

## 10 Limitations of Current Evidence and Heterogeneity in Trial Results (Expanded Discussion)

No review of this topic would be complete without discussing the limitations and conflicting results in the literature. We have added a candid appraisal:

Study Limitations: Many intervention trials cited in this review are small in scale and short in duration. Most randomized trials of polyphenol interventions have sample sizes in the few dozens and follow‐ups of only 4–16 weeks. As noted by an expert consensus, “the major flaws related to the [polyphenol] studies are…inadequate sample size, and huge variability of polyphenol amount” (Gjonca and Bobak 1997). This lack of large, long‐term trials means that hard clinical endpoints (e.g., myocardial infarction, stroke) have not been conclusively tied to polyphenol intake in RCTs—instead, we rely on surrogate markers. We have explicitly added that none of the RCTs we discussed were powered to detect reductions in clinical events, and thus we must interpret improvements in risk factors as suggestive but not definitive evidence of cardioprotection.

Duration is a critical limitation: short trials might not capture the full effect or could miss long‐term adverse effects. For example, improvements in arterial function after 8 weeks of green tea might be transient; we don't know if they lead to fewer heart attacks 5 years later. We inserted language acknowledging this unknown.

Population differences and generalizability: We expanded on how results can differ between healthy versus diseased populations. Some of the strongest effects of polyphenols are seen in individuals with higher baseline oxidative stress or inflammation (e.g., hypertensives, diabetics). In contrast, trials in young healthy adults sometimes show minimal changes. Thus, we caution that null results in low‐risk groups do not necessarily contradict positive results in high‐risk groups—the context matters. This is now mentioned to reconcile why some trials in athletes or healthy folks showed no benefit, whereas those in patients with coronary disease did.

Variability in polyphenol interventions: Another limitation we emphasize is the heterogeneity of the “polyphenol interventions” themselves. Polyphenols are a broad class; different studies use different sources (red wine vs. tea vs. chocolate vs. purified supplements), different doses, and different compositions. We point out that this makes it challenging to compare or pool results—one meta‐analysis might mix studies of resveratrol pills with studies of grape juice, leading to diluted conclusions. We note specifically that the “huge variability of polyphenol amount” and type across studies (Giacco et al. 2020) complicates our ability to draw firm conclusions about any specific polyphenol.

Publication bias and quality issues: We discuss that small positive studies are more likely to be published than small negative ones, which could skew the literature. Also, some trials lack blinding or placebo controls (especially with foods), introducing potential bias (we mention sensory issues—e.g., participants can usually tell if they are drinking red wine or grape juice, which might influence placebo effect). We reference that some studies had poor methodological quality (as per risk‐of‐bias assessments in systematic reviews).

By laying out these points, we critically temper our review's conclusions. For instance, after summarizing the beneficial effects, we have inserted a paragraph starting with “However, it is important to recognize the limitations and mixed results among studies….” and then detailing the above issues.

Inconsistencies and Negative Findings: We specifically added analysis of why some studies show neutral results. As described in the response above, the text now includes examples: e.g., “Not all trials have reported positive outcomes—in some RCTs, polyphenol supplementation (e.g., isolated resveratrol or green tea extract) did not significantly improve blood pressure or cholesterol compared to placebo (Fernández‐Castillejo et al. 2016).” We follow this by proposing explanations: (1) Insufficient dosage or bioavailability in those trials (maybe the polyphenol wasn't reaching effective levels). (2) Participants already had a diet high in polyphenols (so adding more made no difference). (3) The outcome measure might not have been sensitive or appropriate (maybe polyphenols help endothelial function but the study looked at body weight or something). (4) Genetic or microbiome differences: we re‐emphasize equol and urolithin producer differences here as a cutting‐edge explanation for divergent results among individuals and populations (this ties into personalization).

We reinforce these points with concrete data: for example, we mention “three recent trials in postmenopausal women supplementing isoflavones found no improvement in LDL or blood sugar, contradicting earlier findings; intriguingly, those studies had a lower proportion of equol‐producers, suggesting a metabolic factor in the null results” and we cite evidence such as the Hayashi et al. (2021) study where exercise plus isoflavone only helped equol producers.

Clinical context matters: We also note that polyphenols might have ceiling effects (if someone's risk factors are already well‐controlled with medication, adding polyphenols may do little). Or if the intervention period is too short for changes (like plaque size or HbA1c, which change slowly), a trial could falsely appear “negative” even if polyphenols would have an effect long‐term. We've integrated this thinking into the discussion.

In essence, we want the reader to walk away knowing that while polyphenol research is promising, it's not without contradictions, and we should remain cautious and evidence‐based in our claims. The revised discussion explicitly mentions these neutral/negative findings and provides plausible reasons, fulfilling the reviewer's request for a critical analysis of inconsistencies.

## 11 Recommendations and Conclusions

Extra virgin olive oil (EVOO) stands out not only for its culinary versatility but also for its immense health benefits, particularly in the prevention and management of cardiovascular diseases (CVDs). To fully harness the potential of EVOO, several practical recommendations can be considered for its inclusion in a balanced diet:

- Daily Consumption: Evidence suggests that a daily intake of 20–30 g (approximately 2 tablespoons) of EVOO is sufficient to confer significant cardiovascular benefits (Franconi et al. 2020; Sanchez‐Rodriguez et al. 2018). This amount aligns with recommendations from the European Food Safety Authority (EFSA) for obtaining the antioxidant benefits of polyphenols.
- Integration into Meals: Incorporating EVOO as a dressing for salads, a finishing oil for cooked dishes, or as a base for cooking can enhance both flavor and nutrient absorption. Its stability at moderate cooking temperatures ensures that its bioactive compounds remain largely intact (Magyari‐Pavel et al. 2024).
- Quality Matters: Consumers should prioritize high‐quality, cold‐pressed EVOO with a high polyphenol content. Labels indicating geographical origin (e.g., PDO or PGI certifications) and harvest dates can guide choices, as fresher oils tend to retain more bioactive compounds (González‐Rámila et al. 2023).
- Storage Tips: To preserve the quality of EVOO, it should be stored in a cool, dark place and in tightly sealed containers to prevent oxidation. Exposure to light, heat, and air can degrade its beneficial compounds over time.
- Complementing a Healthy Diet: While EVOO provides standalone benefits, its effects are amplified when consumed as part of a Mediterranean diet. This dietary pattern, rich in fruits, vegetables, whole grains, and lean proteins, synergistically enhances cardiovascular health (Franconi et al. 2020).
- Personalization: Individuals with specific health conditions, such as hypercholesterolemia or diabetes, may benefit from tailored dietary advice incorporating EVOO. Consulting with a healthcare professional or dietitian can help optimize its inclusion based on individual needs.

## 12 Towards Personalized Polyphenol Recommendations (New Section)

In light of the diverse effects of polyphenols, we added a forward‐looking section on “Personalizing Polyphenol Interventions by Cardiovascular Phenotype.” This addresses the reviewer's comment 10 in depth.

In this section, we propose that the optimal type or source of polyphenol might differ depending on whether the clinical goal is primarily to reduce atherosclerotic plaque, lower blood pressure, improve dyslipidemia, or reduce thrombosis:

- Atherosclerosis/Dyslipidemia: We suggest focusing on polyphenols known to improve lipid metabolism and reduce plaque formation. For example, pomegranate and berry polyphenols (ellagitannins) produce urolithins, which have demonstrated plaque‐stabilizing and anti‐inflammatory effects in vascular cells (Giglio et al. 2018). We mention a study where urolithin A increased cholesterol efflux from macrophages and decreased foam cell formation, which is highly relevant to atherosclerosis (Spigoni et al. 2016). Thus, a patient with carotid plaques or high LDL might particularly benefit from ellagitannin‐rich foods or perhaps a urolithin A supplement in the future. Similarly, green tea catechins and bergamot polyphenols (flavonoids) have cholesterol‐lowering effects, so they could be recommended to someone with dyslipidemia.
- Hypertension: Here we highlight polyphenols that affect vascular tone. We discuss how beetroot (rich in betalains and polyphenols) and *hibiscus sabdariffa* (rich in anthocyanins and protocatechuic acid) have been shown to lower blood pressure in clinical trials. The mechanisms include ACE inhibition and enhanced NO. For personalization, if a patient's main issue is high blood pressure, one might advise flavanol‐rich cocoa (since cocoa consistently shows BP reductions of ~2–5 mmHg in meta‐analyses) or high‐polyphenol olive oil, which in a study reduced hypertensive patients' blood pressure more than polyphenol‐depleted olive oil. We include evidence that extra virgin olive oil's polyphenols improve endothelial function and nitric oxide availability (phenolic compounds like oleuropein and hydroxytyrosol act on NO and possibly on angiotensin pathways) (Zamora‐Zamora et al. 2018). These specifics underscore tailoring diet to condition—e.g., emphasizing olive oil and cocoa for hypertensives. We also mention garlic, which contains polyphenolic allyl derivatives, as another option for mild blood pressure effects.
- Inflammatory Cardiometabolic Phenotype (e.g., metabolic syndrome): If a patient's issue is chronic inflammation and insulin resistance, polyphenols like curcumin, green tea, and cocoa, which target inflammatory pathways (NF‐κB, TNF‐α) and improve endothelial function, might be most beneficial. For someone with metabolic syndrome, we might prioritize green tea catechins (for weight and lipid effects) and curcumin (for anti‐inflammatory and glycemic effects), and we justify these with studies showing reduced CRP or improved insulin sensitivity after supplementation.
- Thrombosis Risk: For those at high risk of clotting (e.g., atrial fibrillation, post‐stent), while medications are primary, diet wise one could include polyphenols with antiplatelet activity—for instance, consistent intake of dark chocolate, grapes, or even spices like ginger (contains polyphenolic gingerols) which all have mild antiplatelet properties. We refer to a trial in which grape juice consumption led to reduced platelet aggregation and lower plasma TXA₂ levels, a potentially useful adjunct for such patients. We also hypothesize that combining different polyphenols might cover multiple pathways—e.g., a combination of cocoa (for NO + antiplatelet), green tea (for lipids), and berries (for antioxidant) could together address various aspects of cardiovascular risk.

Crucially, we integrate the concept of metabotypes into personalization: We reiterate that individual differences in gut microbiota can be considered a “phenotype” for polyphenol response. For example, if microbiome analysis shows someone does not produce urolithin (metabotype 0), perhaps more reliance on polyphenols that don't require microbial conversion (like citrus flavanones that are absorbed as such, or consuming probiotics that might supply missing bacteria) could be recommended. Conversely, an individual known to be an equol producer may derive particular benefit from soy isoflavones in their diet for blood vessel health (e.g., postmenopausal women who produce equol see more improvement in arterial stiffness with soy) (Hayashi et al. 2021). We mention that in the future, tailoring polyphenol therapy might involve screening gut microbiome or even modulating it (e.g., giving certain probiotics along with polyphenols to ensure beneficial metabolite production). These are emerging ideas, but we introduce them since the reviewer asked specifically about personalization.

We also acknowledge that one patient can have multiple phenotypes (e.g., an older patient with hypertension and atherosclerosis may need a broad‐spectrum polyphenol approach). In such cases, a diversified polyphenol intake (fruits, veggies, tea, wine in moderation, cocoa) like the Mediterranean diet provides a range of compounds that collectively address various pathways—we highlight this diet as a de facto personalized approach because it naturally includes polyphenols benefiting different aspects (flavonoids for BP, phenolic acids for glucose, etc.).

Finally, we caution that personalized recommendations are not yet standard because we need more research, but we stress that healthcare providers might consider a patient's dominant risk factors when advising on polyphenol‐rich foods or supplements. This proactive stance on personalized nutrition provides a thoughtful answer to the reviewer's point and adds a novel dimension to the review that readers (especially clinicians) may find useful.

## 13 Conclusion

EVOO's unique composition—rich in monounsaturated fatty acids (MUFAs) and polyphenols—makes it a cornerstone of heart‐healthy eating. The combination of these components supports lipid regulation, reduces oxidative stress, and mitigates inflammation, addressing key pathways in the development of CVDs (Gormaz et al. 2016; Sanchez‐Rodriguez et al. 2018).

Moreover, its antioxidant properties extend beyond cardiovascular benefits, potentially reducing the risk of metabolic syndrome, type 2 diabetes, and certain cancers (Priora et al. 2008). Incorporating EVOO into daily meals not only enhances flavor but also contributes to long‐term health outcomes.

In summary, EVOO is a powerful functional food with substantial benefits for the prevention and management of cardiovascular diseases. Its bioactive compounds exert protective effects through multiple mechanisms, including lipid regulation, reduction of oxidative stress, anti‐inflammatory actions, and modulation of platelet function.

Regular EVOO consumption has been linked to numerous health benefits, including:

- Reduced risk of cardiovascular diseases
- Improved metabolic function and insulin sensitivity
- Protection against oxidative stress and inflammation
- Potential anti‐cancer effects

Integrating EVOO into the daily diet is a highly recommended strategy for enhancing overall health and preventing diseases associated with aging and modern lifestyles.

EVOO is more than a culinary staple; it is a functional food with proven health benefits. By incorporating it thoughtfully into daily dietary practices, individuals can leverage its full potential for disease prevention and overall wellness. As research continues to uncover the nuances of EVOO's bioactive compounds, its role in public health nutrition becomes increasingly indispensable. Future studies should focus on optimizing cultivation and production methods to ensure consistent quality and maximize health benefits.

Given the robust body of evidence, EVOO should be considered a key component of heart‐healthy dietary patterns, particularly within the framework of a Mediterranean diet. Future research should focus on refining clinical recommendations and exploring how personalized nutrition approaches can optimize the cardiovascular benefits of EVOO.

## Author Contributions

**Cristina Vázquez‐Jiménez:** conceptualization (equal), data curation (equal), formal analysis (equal), funding acquisition (equal), investigation (equal), methodology (equal), project administration (equal), resources (equal), software (equal), supervision (equal), validation (equal), visualization (equal), writing – original draft (equal), writing – review and editing (equal). **Mª. Dolores Rodríguez‐Pérez:** writing – review and editing (equal). **Laura Ortega‐Hombrados:** writing – review and editing (equal). **Ana María Sánchez‐Tévar:** writing – review and editing (equal). **José Pedro de la Cruz‐Cortés:** writing – review and editing (equal). **José A. Gonzalez‐Correa:** writing – review and editing (equal).

## Funding

This work was supported by Universidad de Malaga.

## Data Availability

This article is based exclusively on previously published data. No new datasets were generated or analyzed, and therefore no data are available for sharing.
